# What shapes cognitions of climate change in Europe? Ideology, morality, and the role of educational attainment

**DOI:** 10.1007/s13412-021-00745-7

**Published:** 2022-01-12

**Authors:** Heinz Welsch

**Affiliations:** Department of Economics, University of Oldenburg, 26111 Oldenburg, Germany

**Keywords:** Climate change cognition, Identity-protective cognition, Ideological identity, Moral identity, Moral foundations, Educational attainment, D91, Q54

## Abstract

Cognitions about climate change are of critical importance for climate change mitigation as they influence climate-relevant behaviors and the support of climate policy. Using about 30,000 observations from a large-scale representative survey from 23 European countries, this study provides two major findings. First, important policy-relevant climate change cognitions do not only differ by individuals’ ideological identity (left versus right) but—independently—by their moral identity, that is, the pattern of endorsement of the moral foundations: Care, Fairness, Liberty, Loyalty, Authority, and Purity/Sanctity. In particular, controlling for ideological position, the cognitions that the world climate is changing, that climate change is human-made, and that climate change impacts are bad are significantly negatively related to stronger endorsement of the Authority and Sanctity foundations while being positively related to stronger endorsement of the Loyalty and Fairness foundations. Second, not only the ideology-related cognitive divide but the morality-related divide is larger in individuals with tertiary education, consistent with the idea that individuals with greater science literacy and numeracy use these skills to adjust their cognitions to their group identity. The finding that better education may amplify rather than attenuate the ideology and morality dependence of decision-relevant climate change cognitions sheds doubt on the proposition that better education unambiguously furthers the prospects for climate change mitigation.

## Introduction

Cognitions about climate change are highly relevant for climate change mitigation for at least two reasons (Gifford 2011): First, people unaware of or skeptic about the existence, origins and impacts of climate change are unlikely to take measures to curb greenhouse gas emissions or support public policies to protect the climate. Second, even among those who are aware of the problem, a lack of knowledge about the cause and extent of climate change may lead to ignorance about which (individual and collective) actions are available and how effective different actions are. Sound knowledge about climate change and the options for ameliorating is thus an important precondition for effective climate policy.

Action-relevant cognitions about climate change are shaped by several factors. An obvious source of more accurate knowledge about climate change is better education. Indeed, educational attainment has long been identified as a consistent predictor of environment-related perceptions and concerns (e.g. Dietz et al. 1998), and a meta-analysis of close to 200 polls and academic studies has revealed that education is one of the strongest correlates of the belief in climate change (Hornsey et al. 2016). More recently, cognitions about the existence, origins and impacts of climate change have been found to display a strong left-right ideological divide in many countries, with adherents to the left expressing greater belief in and concern about climate change than adherents to the right (e.g. McCright et al. 2015, McCright et al. 2016, Hornsey et al. 2016, Hornsey et al. 2018). Indeed, political ideology and affiliation are stronger predictors of climate change belief than any other demographic variable (Hornsey et al. 2016).

The psychological mechanism behind the association between ideological identity and climate change beliefs is identity-protective cognition (Kahan et al. 2007), that is, people adjust their beliefs and world views to their personal and social identities in order to minimize cognitive dissonance (Festinger 1957). The techniques employed in forming identity-protective cognitions include individuals’ differentially attending to (through selective exposure or avoidance) and/or processing (through motivated reasoning) information (e.g. Garrett et al. 2011, Kunda 1990, respectively) in a way that agrees with their values and world views. Due to identity-protective cognition, division in terms of ideological position may translate into division of climate change beliefs.

As shown by Kahan et al. (2017a), politically polarized views on science in the U.S. are better explained by identity-protecting cognitive strategies than by the competing hypothesis of deficits in the public’s capacity to comprehend scientific evidence. In addition – contrary to conventional expectations – ideology-dependence of climate change cognitions has been found to be stronger rather than weaker in individuals with greater science literacy and numeracy, on the grounds that these abilities facilitate adjustment of beliefs to identity through selection and processing of information (Kahan et al. 2017a).

Considering that science literacy and numeracy may be related to general educational attainment, this finding suggests that a higher level of education may foster rather than attenuate the identity-dependence of climate change cognitions. Consistent with this view, Czarnek et al. (2020) found the ideological divide of climate change cognitions in developed countries to be increasing in individuals’ years of education, whereas the evidence is ambiguous with respect to less developed countries.

While the bulk of the literature on identity-protective climate change cognitions focused on ideological identity (political affiliation or position on the left-right scale), one recent paper (Welsch 2021) has studied the relationship between moral identity and climate change cognitions. Drawing on so-called moral foundations theory (Haidt and Joseph 2007, Graham et al. 2011, Haidt 2012), the paper found significant relationships between beliefs which foster climate friendly behaviors and the endorsement of universalist (individual-focused) -- as opposed to parochial (group-focused) -- moral values. Specifically, individuals hold stronger beliefs that climate change has bad impacts the more they endorse the moral values of Fairness and Liberty (universalist values) and the less they endorse Authority and Sanctity (parochial values). Importantly, these relationships hold even when controlling for ideological identity (position on the left-right scale).

It should be noted that much of the work on ideology-dependent cognition, motivated reasoning, and the moderating role of science literacy and numeracy focused on North American populations (e.g., Kahan et al. 2007, Kahan et al. 2017a). Analyzing data from 25 developed and emerging countries, Hornsey et al. (2018) found relationships between climate skepticism and 5 ideological variables to be statistically significantly stronger in the U.S. than in the pool of 24 other nations. The relationships also tended to be stronger in 5 English speaking nations (other than the U.S.) than in the 24-nation pool, but not significantly so. At the level of individual countries, the relationship between climate skepticism and more right-leaning ideology was positive in 19 out of 25 countries (significantly so in 7 countries). While there does not seem to be evidence on the moderating role of science literacy and numeracy in the cognition-ideology relationship outside the U.S. (Kahan et al. 2017a), Czarnek et al. (2020) found a significant moderating role of the related notion of educational attainment in a set of 22 European countries. With respect to the relationship between climate change cognitions and moral identity, a moderating role for science literacy and numeracy or education has not been studied as yet.

The present paper studies the role for climate change cognitions of ideological identity, moral identity, and cognitive ability jointly. It augments the literature on climate change cognitions, identity, and cognitive skills and abilities in several ways. First, it extends the evidence on the ideology-cognition-ability relationship (Kahan et al. 2017a) from a small U.S. sample to a large representative European sample. Second, in focusing on the morality-cognition nexus it studies a larger set of climate change cognitions than previously considered (Welsch 2021). Third, using educational attainment as a measure of cognitive ability, it studies for the first time the role of cognitive ability as a potential moderator of the morality-cognition relationship. Finally, it discusses climate policy conclusions, drawing on the notion of the “tragedy of the science communication commons” (Kahan 2017, Kahan et al. 2017b).

The empirical analysis uses about 30,000 observations for 23 European countries from Round 8 of the European Social Survey (ESS) and involves four climate change cognitions (the world climate is changing; climate change is caused by human activities; climate change has bad impacts; reducing personal-level energy use can reduce climate change), individuals’ placement on the left-right scale, and individuals’ endorsement of six moral foundations (Care, Fairness, Liberty, Loyalty, Authority, and Purity/Sanctity).

### Key findings and conclusions

The key findings and conclusions can be summarized as follows:

- Action-relevant climate change cognitions of European citizens are shaped not only by ideological identity (left-right) but by moral identity (universalist-parochial), where more right-leaning individuals and those with a more parochial (group-focused) morality display greater climate skepticism than more left-leaning individuals and those with a more universalist (individual-focused) morality.
- In contrast to popular views, more education is not on its own a solution to climate denial, as a higher level of education is associated with higher rather than lower ideology and morality dependence of important climate change cognitions.
- To minimize harmful identity dependence, climate cognitions and climate policy should be sheltered from being fused with antagonistic policy issues such as state interference or national sovereignty.
- To attenuate the morality-related divide, climate change mitigation should be (re)framed such as to establish an accord between mitigation and the moral values that are presently associated with climate skepticism (for instance framing degradation of the climate system as a violation of Purity/Sanctity).

### Detailed results

In more detail, multiple regression analysis reveals that all cognitions studied are significantly negatively related to a more right-leaning ideological position and significantly positively related to stronger endorsement of Fairness, whereas all but one cognitions are significantly negatively related to stronger endorsement of Authority and Sanctity. Similar to the literature on the role of science literacy, the paper finds that the ideology dependence of the cognitions studied is significantly stronger in individuals with a tertiary education (that is, a bachelor degree or higher). In addition to the ideology-cognition relationships, 9 of the 24 morality-cognition relationships are also significantly stronger in individuals with tertiary education. In particular, the cognitions that climate change is human-made and that climate change impacts are bad are more morality dependent in individuals with better education than in less educated people. In contrast to the latter cognitions, the belief that the climate is changing is—on balance—less morality dependent in better educated than in less well educated people. The level of education per se is positively related to the cognitions that the climate is changing, that climate change is human-made, and that climate change impacts are bad. The role of better education in shaping climate change cognitions is thus ambiguous as better education per se improves climate knowledge but tends to amplify biases in the relevant cognitions that result from identity-protective information selection and processing.

Observing that Fairness falls into the category of universalist (individual-focused) moral foundations whereas Authority and Sanctity are constitutive of a parochial (group-focused) morality (Graham et al. 2011, Haidt 2012), it can be noted that in addition to the familiar ideology-related divide in climate change cognitions there is a divide between adherents to a universalist and a parochial morality and that the level of education tends to widen rather than narrow both types of divide.

### Organization of the paper

The remainder of the paper is organized as follows. The “Method” section presents the data and empirical strategy. The “Results” section presents the results. The “Discussion and climate policy conclusions” section offers a discussion and some climate policy conclusions.

## Method

### Data and variables

The data used in the empirical analysis are taken from the European Social Survey (ESS) (see www.europeansocialsurvey.org). The ESS is a cross-sectional, multi-country survey covering over 30 nations. ESS data are obtained using random (probability) samples, where the sampling strategies are designed to ensure representativeness and comparability across European countries. I use data from Round 8 (2016) because it includes a “Climate Change” module that offers data for climate change cognitions, along with ideological position, endorsement of the moral foundations, educational attainment, and a set of sociodemographic control variables. ESS Round 8 covers the following countries: Austria, Belgium, Czech Republic Estonia, Finland, France, Germany, Hungary, Ireland, Israel, Iceland, Italy, Lithuania, Netherlands, Norway, Poland, Portugal, Russian Federation, Sweden, Slovenia, Spain, Switzerland, and the UK. The total number of valid cases is 44,387. The number of observations available for regression analysis amounts to about 30,000, depending on the specific specification.

### Dependent variables

The dependent variables (climate change cognitions) are the degrees of agreement to the propositions that the world climate is changing, that climate change is human-made, that climate change has bad impacts, and that changing one’s own energy consumption is effective in mitigating climate change. The respective survey questions, coding, and summary statistics are as follows:

- *World Climate is Changing*: Do you think the world’s climate is changing? 1 = “definitely not changing” to 4 = “definitely changing” (original coding reversed for empirical analysis). Mean = 3.48, SD = 0.69.
- *Climate Change Human-Made*: Do you think that climate change is caused by natural processes, human activity, or both? 1 = “entirely by natural processes,” 2 = “mainly by natural processes,” 3 = “about equally by natural processes and human activity,” 4 = “mainly by human activity,” 5 = “entirely by human activity.” The response “I don’t think climate change is happening” was omitted. Mean = 3.42, SD = 0.80.
- *Climate Change Impacts Bad*: How good or bad do you think the impact of climate change will be on people across the world? 0 = “extremely good” to 10 = “extremely bad” (original coding reversed for empirical analysis). Mean = 6.74, SD = 2.20.
- *Individual Mitigation Effective*: How likely do you think it is that limiting your own energy use would help reduce climate change? 0 = “not at all likely” to 10 = “extremely likely.” Mean = 4.35, SD = 2.65.

### Explanatory variables

The main explanatory variables are an indicator of whether an individual has tertiary education, the position on the left-right scale, and the degrees of endorsement of the moral foundations. The respective survey questions, coding, and summary statistics are as follows:

- *Tertiary*: What is your highest level of education? 1 = less than lower secondary, 2 = lower secondary, 3 = lower tier upper secondary, 4 = upper tier upper secondary, 5 = advanced vocational (sub-degree), 6 = lower tertiary education (BA level), 7 = higher tertiary education (MA level or higher). The first five levels were coded as zero, and the last two levels were coded as 1. Mean = 0.24, SD = 0.37.
- *Right*: In politics, people sometimes talk of “left” and “right.” Using this card, where would you place yourself on this scale, where 0 means the left and 10 means the right? Mean = 5.16, SD = 2.24.
- Moral foundations: “Now I will briefly describe some people. Please tell me how much each person is or is not like you.” 1 = “not at all like me” to 6 = “very much like me” (original coding reversed for empirical analysis).
- *Care*: “It is very important to her/him to help the people around her/him. She/he wants to care for their wellbeing.” Mean = 3.80, SD = 1.01.
- *Fairness*: “She/he thinks it is important that every person in the world should be treated equally. She/he believes everyone should have equal opportunities in life.” Mean = 3.82, SD = 1.08.
- *Liberty*: “It is important to her/him to make her/his own decisions about what she/he does. She/he likes to be free and not depend on others.” Mean = 3.82, SD = 1.10.
- *Loyalty*: “It is important to her/him to be loyal to her/his friends. She/he wants to devote herself/himself to people close to her/him.” Mean = 4.04, SD = 0.95.
- *Authority*: “She/he believes that people should do what they’re told. She/he thinks that people should follow rules at all times, even when no-one is watching.” Mean = 2.77, SD = 1.40.
- *Sanctity*: “Tradition is important to her/him. She/he tries to follow the customs handed down by her/his religion or her/his family.” Mean = 3.82, SD = 1.05.
- The ESS items used to measure endorsement of Care, Fairness, Liberty, Loyalty, Authority, and Sanctity do not explicitly refer to moral foundations theory. Rather, the correspondence of the survey items to the moral foundations relies on the formulations used in the survey. Welsch (2020) shows that the moral foundations proxied this way have very similar properties (correlations with each other and with sociodemographic variables) as variables obtained from the “Moral Foundations Questionnaire” (Graham et al. 2011). It may also be noted that some of the survey questions explicitly involve a differentiation between individual-focused (universalistic) and group-focused (parochial) morality. This is most salient with respect to Fairness and Loyalty, the former referring to “every person in the world,” whereas the latter refers to “people close.”

### Control variables

Control variables are age (years), gender (0 = male, 1 = female), net household income (deciles), number of people living in the household, domicile (1 = “big city” to 5 = “countryside”), and subjective general health (1 = “very good” to 5 = “very bad”).

### Empirical strategy

The regression equations take the following general form:

$${Cognition}_{i}=const+{b}_{1}{{Right}_{i}+ b}_{2}{Morals}_{i} +{b}_{3}{{Tertiary}_{i}+{{b}_{4}Right}_{i}*{Tertiary}_{i}+{{b}_{5}Morals}_{i}*Tertiary+b}_{6}{Controls}_{i}+{error}_{i}$$

where *Cognition*_*i*_ denotes any of individual *i*’s climate change cognitions described in the “Data and variables” subsection and *Morals*_*i*_ and *Controls*_*i*_ are vectors comprising the moral foundations and the control variables, respectively.

The regression equations were estimated using ordinary least squares since ordered probit or logit estimators yield ambiguous marginal effects in interaction models (Ai and Norton 2003).

## Results

### Correlations

The correlations between the variables of interest are shown in Table 3 in the Appendix. The cognition that individual mitigation behavior is effective displays no significant correlation with the level of education and very low correlations with both ideological position and endorsement of the moral foundations (*r* < 0.1). The cognitions that the world climate is changing, that climate change is human-made, and that climate change impacts are bad are positively correlated with the level of education, but the correlations are very low (*r* < 0.1). These cognitions are negatively correlated with a more right-leaning ideological position (*r* = −0.098 to −0.127) and with the moral foundations of Authority and Sanctity while being positively correlated with the moral foundations of Care, Fairness, Liberty, and Loyalty. Some of these positive correlations are of a similar or larger magnitude than the negative ideology-cognition correlations. The correlations between right-leaning ideological position and endorsement of the moral foundations are very low (*r* < 0.1) except for Fairness (*r* = −0.145) and Sanctity (*r* = 0.132). Ideological position and moral identity are thus fairly independent of each other.^1^

The correlations suggest that ideological position and endorsement of the moral foundations may play independent roles as predictors of climate change cognitions, if any. This expectation was checked by means of multivariate regression analysis.

### Regression results

Table 1 displays the results of multivariate regressions with several climate change cognitions as the dependent variables: the beliefs that the world climate is changing, that climate change is human-made, that climate change impacts are bad, and that individual mitigation behaviors are effective. As seen in the first row, all four beliefs are significantly negatively related to a more right-leaning ideological position. In addition, as shown by the interaction terms between ideological position and tertiary education, the negative ideology-cognition relationships are significantly stronger in individuals with tertiary education (bachelor degree or higher). By contrast, tertiary education per se is significantly positively related to the beliefs that the world climate is changing, that climate change is human-made, and that climate change impacts are bad, while not being significantly related to the belief that individual mitigation behaviors are effective.^2^

**Table 1:** Regression results

	World climate is changing	Climate change human-made	Climate change impacts bad	Individual mitigation effective
Right Right*Tertiary	−0.02*** (9.74) −0.01*** (2.73)	−0.03*** (11.80) −0.01** (1.93)	−0.09*** (14.14) −0.03** (2.27)	−0.02** (2.04) −0.03** (1.73)
Care Care*Tertiary	0.05*** (9.03) −0.01*** (8.06)	0.01 (0.97) 0.00 (0.09)	0.01 (0.67) 0.05* (1.52)	0.21*** (9.68) 0.00 (0.20)
Fairness Fairness*Tertiary	0.05*** (11.56) 0.00 (0.56)	0.05*** (9.47) 0.02** (1.80)	0.19*** (12.56) 0.03 (1.03)	0.12*** (6.75) 0.08 ** (2.17)
Liberty Liberty*Tertiary	0.02*** (5.46) −0.01* (1.63)	0.01 (0.70) 0.00 (0.48)	0.08*** (5.76) −0.08* (1.36)	−0.08*** (4.65) −0.01 (0.26)
Loyalty Loyalty*Tertiary	0.04*** (6.63) −0.01 (0.95)	0.02*** (3.46) 0.02* (1.61)	0.13*** (7.17) 0.06** (1.67)	−0.10*** (4.55) 0.06 (1.11)
Authority Authority*Tertiary	−0.03*** (9.03) 0.01* (1.43)	−0.01*** (2.87) −0.03*** (3.75)	−0.06*** (5.86) −0.05*** (2.43)	0.04*** (2.73) 0.00 (0.18)
Sanctity Sanctity*Tertiary	−0.02*** (4.71) −0.01* (1.52)	−0.02*** (3.89) 0.00 (0.50)	−0.05*** (4.20) −0.04** (1.86)	0.07*** (5.22) 0.03 (1.06)
Tertiary	0.12*** (3.85)	0.09*** (2.92)	0.45*** (6.43)	1.10 (0.69)
Controls	Yes	Yes	Yes	Yes
Constant	3.76	3.84	7.79	5.21
*R*^2^	0.046	0.036	0.051	0.020
Observations	30628	29860	29540	29781

OLS regressions; t-statistics in parentheses based on country-clustered standard errors. *p<0.1, **p<0.05, ***p<0.01. All regressions control for age, gender, household income, household size, subjective general health, and domicile (urban-rural scale)

Turning to the moral foundations, the following findings stand out:

- The cognition that the world climate is changing is significantly positively related to stronger endorsement of the Care, Fairness, Liberty, and Loyalty foundations and significantly negatively related to stronger endorsement of the Authority and Sanctity foundations. With respect to Care, Liberty, and Authority, the morality-cognition relationships are significantly weaker in better educated people, while with respect to Sanctity, the relationships are significantly stronger in better educated people.
- The cognition that climate change is human-made is significantly positively related to stronger endorsement of the Fairness and Loyalty foundations and significantly negatively related to stronger endorsement of the Authority and Sanctity foundations. With respect to Fairness, Loyalty, and Authority, the morality-cognition relationships are significantly stronger in better educated people, whereas education does not significantly moderate the other morality-cognition relationships.
- The cognition that climate change impacts are bad is significantly positively related to stronger endorsement of the Fairness, Liberty, and Loyalty foundations and significantly negatively related to stronger endorsement of the Authority and Sanctity foundations. With respect to Care, Loyalty, Authority, and Sanctity, the morality-cognition relationships are significantly stronger in better educated people, while with respect to Liberty, they are weaker in better educated people.
- The cognition that individual mitigation behaviors are effective is significantly positively related to stronger endorsement of the Care, Fairness, Authority, and Sanctity foundations and significantly negatively related to stronger endorsement of the Liberty and Loyalty foundations. These relationships are not significantly moderated by the level of education, except for the case of Fairness where the morality-cognition relationship is significantly stronger in better educated people.

These results suggest that moral identity plays a role in shaping climate change cognitions, as does ideological identity, and that both types of relationship are moderated by education. The next subsection provides a more concise picture of the morality-cognition relationships.

### Universalist and parochial morality

As was noted in the “Introduction,” the moral foundations can be grouped into individual-focused or universalist foundations in the sense that they refer to all individuals independent of what group they belong to (such as family, neighborhood, region, or nation)—Care, Fairness, and Liberty—and parochial foundations in the sense that they contribute to the stability of an in-group—Loyalty, Authority, and Purity/Sanctity (Graham et al. 2011, Haidt 2012).^3^ Given the character of climate change mitigation as a global public good that benefits everyone (rather than being group-specific), it is instructive to check whether the estimation results differ in terms of the universalist-parochial categorization.

Table 2 displays the sum across universalist and parochial moral foundations, respectively, of the significant coefficients from Table 1. The overall messages are (a) that endorsement of the universalist foundations is positively associated with stronger climate change beliefs, particularly so in individuals with tertiary education and (b) that endorsement of the parochial foundations is almost unrelated with stronger climate change beliefs in individuals without tertiary education and slightly negatively associated with such beliefs in individuals with tertiary education.

**Table 2:** Aggregate morality-cognition relationships

	World climate is changing	Climate change human-made	Climate change impacts bad	Individual mitigation effective	Sum
Universalist	0.12	0.05	0.27	0.25	0.69
Universalist*tertiary	n.s.	n.s.	0.13	0.08	0.23
Parochial	−0.01	−0.01	0.02	0.01	0.01
Parochial*tertiary	n.s.	−0.1	−0.03	n.s.	−0.04
A&S	−0.05	−0.03	−0.11	0.11	−0.08
A&S*tertiary	n.s.	−0.03	−0.09	n.s.	−0.12

The entries are the sum of significant coefficients from Table 1*(n.s.* not significant). Universalist = Care + Fairness + Liberty. Parochial = Loyalty + Authority + Sanctity. A&S = Authority + Sanctity

While these results are broadly consistent with a universalist-parochial dichotomy of the morality-cognition relationship (particularly so in better educated people), Table 1 has revealed that the morality-cognition relationship tends to be different with respect to Loyalty (positive) than with respect to Authority and Sanctity (negative). When we focus on the latter two foundations, we find that they are negatively associated with the first three beliefs and more strongly so in better educated people (Table 2, last two rows). In particular, the beliefs that climate change is human-made and that climate impacts are bad are strongly negatively related to endorsement of the Authority and Sanctity foundations in individuals with tertiary education. As the last column of Table 2 reveals, a 1-unit increase in endorsement of the universalist moral foundations among individuals with tertiary education goes with a change of pro-climate beliefs by 0.31 (= (0.69 + 0.23)/3) points on average, whereas a 1-unit increase in endorsement of Authority and Sanctity goes with a change of pro-climate beliefs by −0.10 (= −(0.08+0.12)/2) points on average.

## Discussion and climate policy conclusions

### Summary and interpretation of findings

Using a large-scale representative survey from 23 European countries, the analysis has revealed that important policy-relevant climate change cognitions do not only differ by individuals’ level of education (e.g., Dietz et al. 1998) and ideological identity (e.g., McCright et al. 2015, McCright et al. 2016, Hornsey et al. 2016, Hornsey et al. 2018**)** but—independently—by their moral identity as described by moral foundations theory. In particular, the cognitions that the world climate is changing, that climate change is human-made, and that climate change impacts are bad are significantly negatively related to stronger endorsement of the Authority and Sanctity foundations while being positively related to stronger endorsement of the Loyalty and Fairness foundations. In addition, the cognition that individual mitigation behavior is effective is positively related to Fairness (as well as Care). These findings corroborate the evidence presented by Welsch (2021) and extend it to a larger set of climate change cognitions and to a larger set of countries.^4^

Similar to previous research that found the ideological divide in climate change cognitions to be larger in individuals with greater science literacy and numeracy (e.g., Kahan et al. 2012), the present analysis found the ideology-cognition relationship to be stronger in individuals with better (that is, tertiary) education. While this is consistent with previous findings (e.g., Czarnek et al. 2020), a novel contribution of the present work is that not only the ideology-cognition relationship but the morality-cognition relationships are stronger in better educated people. This applies in particular to the cognitions that climate change is human-made and that climate change impacts are bad. These latter beliefs are not only negatively related to stronger endorsement of Authority and Sanctity, but the negative relationships are stronger in individuals with tertiary education,

With respect to the morality-cognition relationships, two comments are in order. First, the relationships tend to differ between universalist morality (positive relationship to pro-climate cognitions) and parochial morality (negative relationship to pro-climate cognitions). However, the dichotomy is not clear-cut. In particular, while the parochial values Authority and Sanctity are negatively related to the beliefs of existence, human origin, and negative impact of climate change, the parochial value Loyalty is positively related to those beliefs.

Second, it is not necessarily the case that Authority and Sanctity are associated with incorrect and Care and Fairness are associated with correct beliefs. While this is true with respect to the beliefs concerning the existence, origin, and impacts of climate change, Care and Fairness are also associated with the belief that individual mitigation behaviors are effective in reducing climate change—a belief that is at least questionable in view of each individual’s small contribution to climate change.^5^

With respect to the interaction terms between ideology and morality, on the one hand, and education, on the other, it is clear that they can be read in two ways. They may indicate how education affects the ideology-cognition and morality-cognition relationships, or how ideology and morality, respectively, affect the education-cognition relationship. With respect to ideology, Czarnek et al. (2020) adopt the latter interpretation, asserting that right-wing ideology attenuates (but not reverses) the positive effect of education on pro-climate change beliefs in more developed countries.

Taking a different perspective, Kahan et al. (2012) and Kahan (2017) argue that better science literacy and numeracy (to which tertiary education arguably contributes) enables individuals to more effectively select and process information in order to align their beliefs to those prevalent in their (ideologically defined) peer group. The argument put forward to support this view relies on the risk of losing the psychic and material support of one’s peers in case of dissenting beliefs on climate change: Since a single person cannot meaningfully affect the climate, the costs of losing peer support exceed the benefit from engaging in mitigation behavior in accordance with insights from climate science. Maintaining group identity and group support is thus the ultimate concern, and better cognitive skills facilitate adjustment of beliefs to identity such as to secure the benefits of group membership.

### Climate policy conclusions

Cognitions about the existence, origins, and impacts of climate change are important determinants of individuals’ mitigation behavior (Welsch 2021), and they are strongly influenced by individuals’ ideological and moral identities. The finding that better education may amplify rather than attenuate the ideology and morality dependence of decision-relevant climate change cognitions is worrying as it sheds doubt on the proposition that better education unambiguously furthers the prospects for climate change mitigation. While better education per se entails a better understanding of climate change issues, the cognitive skills associated with better education may enable individuals with a right-leaning ideological identity and those with a moral identity based on Authority and Sanctity to adjust their beliefs to their identity-related “climate skepticism.”

The persistence of (incorrect) identity-protecting climate change beliefs is an instance of what Kahan (2017) calls the “tragedy of the science communication commons”: It is individually rational for an individual to hold “climate skeptic” beliefs if (i) such beliefs are an essential element of an individual’s group identity, (ii) dissent with group beliefs involves the threat of losing (psychic and material) group support, and (iii) the costs of losing group support exceed the benefit from engaging in mitigation behavior—which is likely since a single individual has little impact on the climate.

Since better education is of limited value in overcoming this “tragedy,” the question arises as to what may be more effective communication strategies. One approach involves sheltering the knowledge-transmission process against disruption by “antagonistic meanings that fuse positions on disputed facts to individuals’ cultural identities” (Kahan 2017). Such meanings—see below—divert individuals, particularly those who are otherwise the most proficient thinkers, from using their reasoning to recognize what science knows and instead redirects it to conforming their beliefs to the ones that predominate in their cultural group (Kahan 2017, Kahan et al. 2017b).

In the case of climate change, such disruption of adequate knowledge transmission may result from the climate issue being (opportunistically) fused with notions such as climate policy involving (excessive) state interference or implying a cut-back on national sovereignty and independence. With respect to state interference, attenuating such concerns may include, for instance, the propagation of market-based instruments rather than command-and-control mitigation policies. With respect to national sovereignty concerns, the framing of climate policy as an opportunity to take a pioneering role or as a “national mission” deserving to take pride in may help.

With respect to moral identity, attenuating the divide may involve reframing climate change mitigation such as to establish an accord (rather than an opposition) between mitigation and the moral values that presently are associated with climate skepticism. That such a strategy may be effective was shown by Feinberg and Willer (2019), who found that framing environmental pollution as a violation of Purity/Sanctity is an effective and persuasive technique for communication across divides and increasing environmental awareness among conservatives. Similarly, related to the moral value of Authority, it was shown (in the context of the COVID-19 pandemic) that appealing to moral duty is an effective way of stimulating containment behaviors that are costly and involve a large gap between individual and social benefits (Quaas et al. 2021).

These findings suggest that the relationships between climate change cognitions and identity unearthed in the present study are not necessarily immutable. By loosening the tie between cognitions and group identity, it may be possible for pro-climate cognitions and behavioral norms to spread between groups, eventually affecting behaviors on large scales. Examples such as the abandonment of foot-binding in China, changed fertility norms, changed norms for indoor smoking, and the declining popularity of animal foods in some western societies (Nyborg et al. 2016) suggest that similarly lowering the identity relevance of beliefs and norms may also be possible in the case of inadequate climate change cognitions and the behaviors they support.

## Appendix

**Table 3:** Correlations

	Tertiary	Right	Care	Fairness	Liberty	Loyalty	Authority	Sanctity	World climate is changing	Climate change human-made	Climate change impacts bad
Right	−0.014*										
Care	−0.003	−0.051**									
Fairness	0.009	−0.145**	0.359**								
Liberty	0.057**	−0.015**	0.286**	0.212**							
Loyalty	0.021**	−0.017**	0.475**	0.316**	0.290**						
Authority	−0.030**	0.082**	0.116**	0.072**	0.007	0.100**					
Sanctity	−0.061**	0.132**	0.208**	0.081**	0.048**	0.185**	0.274**				
World climate is changing	0.035**	−0.115**	0.126**	0.153**	0.104**	0.116**	−0.046**	−0.030**			
Climate change human-made	0.036**	−0.098**	0.049**	0.093**	0.036**	0.058**	−0.046**	−0.055**	0.245**		
Climate change impacts bad	0.057**	−0.127**	0.075**	0.133**	0.077**	0.104**	−0.065**	−0.053**	0.270**	0.264**	
Individual mitigation effective	0.001	−0.020**	0.085**	0.069**	−0.010	0.023**	0.034**	0.055**	0.075**	0.107**	−0.029**

^*^*p* < 0.05, ** *p* < 0.01

## Footnotes

1 Haidt (2012) suggests that left-leaning (liberal) and right-leaning (conservative) individuals endorse the universalist moral foundations in similar ways but liberals endorse the parochial moral foundations less than do conservatives.

2 The control variables female (dummy variable) and income attract significantly positive coefficients; age and poor health attract significantly negative coefficients. The coefficients for household size and domicile (urban-rural scale) are not significant.

3 Purity/Sanctity can be considered to be group-focused since different groups (such as religious denominations or nations) are often characterized by different beliefs or practices being considered as inviolable.

4 Welsch (2021) focused on the cognitions that climate change is bad and that individual mitigation behaviors are effective and on individuals from West European countries.

5 Given that the moral foundations—including Care and Fairness—have emerged as a response to free-rider issues in public good provision (Haidt 2012, Welsch 2020), this is an example of moral values contributing to collective rationality (efficiency) through fostering an incorrect yet “useful” belief.

## References

[CR1] Ai C, Norton EC (2003). Interaction terms in logit and probit models. Econ Lett.

[CR2] Czarnek G, Kossowska M, Szwed P (2020). Right-wing ideology reduces the effects of education on climate change beliefs in more developed countries. Nat Clim Chang.

[CR3] Dietz T, Stern PC, Guagnano GA (1998). Social structural and social psychological bases of environmental concern. Environ Behav.

[CR4] Feinberg M, Willer R (2019). Moral reframing: a technique for effective and persuasive communication across political divides. Social and Personality Psychology Compass.

[CR5] Festinger L (1957). A theory of cognitive dissonance.

[CR6] Garrett RK, Carnahan D, Lynch EK (2011). A turn toward avoidance? Selective exposure to online political information, 2004–2008. Polit Behav.

[CR7] Gifford R (2011). The dragons of inaction: psychological barriers that limit climate change mitigation and adaptation. Am Psychol.

[CR8] Graham J, Nosek BA, Haidt J, Iyer R, Koleva S, Ditto PH (2011). Mapping the moral domain. J Pers Soc Psychol.

[CR9] Haidt J (2012). The righteous mind: why good people are divided by politics and religion.

[CR10] Haidt J, Joseph C, Carruthers P, Laurence S, Stich S (2007). The moral mind: how 5 sets of innate intuitions guide the development of many culture-specific virtues and perhaps even modules. The Innate Mind.

[CR11] Hornsey MJ, Harris EA, Bain PG, Fielding KS (2016). Meta-analyses of the determinants and outcomes of belief in climate change. Nat Clim Chang.

[CR12] Hornsey MJ, Harris EA, Fielding KS (2018). Relationships among conspiratorial beliefs, conservatism and climate scepticism across nations. Nat Clim Chang.

[CR13] Kahan MD, Braman D, Gastil J, Slovic P, Mertz CK (2007). Culture and identity-protecting cognition: explaining the white-male effect in risk perception. J Empir Leg Stud.

[CR14] Kahan, D.M. (2017), Misconceptions, misinformation and the logic of identity-protective cognition, Yale Law & Economics Research Paper No. 575.

[CR15] Kahan DM, Peters E, Wittlin M, Slovic P, Larrimore Ouellette L, Braman D, Mandel G (2012). The polarizing impact of science literacy and numeracy on perceived climate change risks. Nat Clim Chang.

[CR16] Kahan DM, Cantrell Dawson E, Peters E, Slovic P (2017). Motivated numeracy and enlightened self-government. Behavioral Public Policy.

[CR17] Kahan DM, Jamieson KH, Landrum A, Winneg K (2017). Culturally antagonistic memes and the Zika virus: an experimental test. J Risk Res.

[CR18] Kunda Z (1990). The case for motivated reasoning. Psychological Bulletin.

[CR19] McCright AM, Dunlap RED, Marquart-Pyatt ST (2015). Political ideology and views about climate change in the European Union. Environmental Politics.

[CR20] McCright AM, Marquart-Pyatt ST, Shwom RL, Brechin SR, Allen S (2016). Ideology, capitalism, and climate: explaining public views about climate change in the United States. Energy Res Soc Sci.

[CR21] Nyborg K, Anderies JM, Dannenberg A, Lindahl T, Schill C, Maja Schlüter W, Adger N, Arrow KJ, Barrett S, Stephen Carpenter F, Stuart Chapin III, Crépin A, Daily G, Ehrlich P, Folke C, Jager W, Kautsky N, Levin SA, Madsen OJ, Polasky S, Scheffer M, Walker B, Weber EU, Wilen J, Xepapadeas A, de Zeeuw A (2016). Social norms as solutions. Science.

[CR22] Quaas MF, Meya JN, Schenk H, Bos B, Drupp MA, Requate T (2021). Moral suasion and the private provision of public goods: Evidence from the *COVID*-19 pandemic. Environ Resource Econ.

[CR23] Tomasello M (2016). A natural history of human morality.

[CR24] Welsch H (2020). Moral foundations and voluntary public good provision: the case of climate change. Ecological Economics.

[CR25] Welsch H (2021). How climate-friendly behavior relates to moral identity and identity-protective cognition: evidence from the European Social Surveys. Ecological Economics.

[CR26] Welsch H, Kühling J (2017). Pan-European patterns of environmental concern: the role of proximity and international integration. J Environ Stud Sci.

